# Scalable and exhaustive screening of metabolic functions carried out by microbial consortia

**DOI:** 10.1093/bioinformatics/bty588

**Published:** 2018-09-08

**Authors:** Clémence Frioux, Enora Fremy, Camille Trottier, Anne Siegel

**Affiliations:** 1 Univ Rennes, Inria, CNRS, IRISA, Rennes, France; 2 Université de Nantes, LS2N, CNRS, Nantes, France

## Abstract

**Motivation:**

The selection of species exhibiting metabolic behaviors of interest is a challenging step when switching from the investigation of a large microbiota to the study of functions effectiveness. Approaches based on a compartmentalized framework are not scalable. The output of scalable approaches based on a non-compartmentalized modeling may be so large that it has neither been explored nor handled so far.

**Results:**

We present the Miscoto tool to facilitate the selection of a community optimizing a desired function in a microbiome by reporting several possibilities which can be then sorted according to biological criteria. Communities are exhaustively identified using logical programming and by combining the non-compartmentalized and the compartmentalized frameworks. The benchmarking of 4.9 million metabolic functions associated with the Human Microbiome Project, shows that Miscoto is suited to screen and classify metabolic producibility in terms of feasibility, functional redundancy and cooperation processes involved. As an illustration of a host-microbial system, screening the Recon 2.2 human metabolism highlights the role of different consortia within a family of 773 intestinal bacteria.

**Availability and implementation:**

Miscoto source code, instructions for use and examples are available at: https://github.com/cfrioux/miscoto.

## 1 Introduction

The rise of metagenomics through sequencing advances and efficient computational biology techniques has led to a broad range of data and perspectives for unraveling the complexity of ecosystems and elucidating the role of species within microbiomes (Marchesi *et al.*, 2015). Altered traits displayed by some species in axenic cultures confirmed that phenotypes are linked to interactions occurring between an organism and its microbiota (Douglas, 1992; Ridley *et al.*, 2012; Tapia *et al.*, 2016). Therefore, more consideration tends to be given to organisms as parts of communities or interacting actors (Fuentes *et al.*, 2016; KleinJan *et al.*, 2017; Molloy *et al.*, 2017) while exploration of organisms in isolation is acknowledged to be reductionist (Cavaliere *et al.*, 2017). Bioinformatics methods are of great help in understanding, modeling, or suggesting interactions within microbiomes, at several scales and levels of resolution (Li *et al.*, 2016). At a first rough scale, investigations relating to microbial co-occurrence apply to the full sequenced microbiotas (Faust *et al.*, 2012). Then, metabolic models give insights into the precise mechanisms underlying the competition, cooperation or commensalism relationships. They have been widely exploited (van der Ark *et al.*, 2017) using methods ranging from the modeling of meta-organisms with abstracted boundaries (Greenblum *et al.*, 2012) to constraint-based modeling (Heinken *et al.*, 2015b) of small groups of organisms, often called communities (Khandelwal *et al.*, 2013; Stolyar *et al.*, 2007).

Naturally, the resolution of the proposed interactions will be more precise as both the size of the community decreases and the degree of knowledge on its members increases (Johns *et al.*, 2016). In large communities, graph-based analysis of metabolic models allows for a fast and efficient screening of organisms by scoring their competition (Kreimer *et al.*, 2012) or cooperation (Levy *et al.*, 2015) potential. When focusing on a smaller number of organisms, it becomes possible to quantitatively predict the behaviors of the system, which may be either the production of compounds of interest in synthetic biology studies in a controlled system (Großkopf *et al.*, 2014), or simply growth with respect to environmental studies. For instance, (Greenblum *et al.*, 2012) relied on a constraint-based framework to model each species of a community as a compartment that interacts with others. This allows growth to be simulated (Mendes-Soares *et al.*, 2016) possibly in a dynamic way (Zomorrodi *et al.*, 2014), coupled with co-occurrence data (Zelezniak *et al.*, 2015) or with the integration of spatial/temporal constraints (Bauer *et al.*, 2017; Harcombe *et al.*, 2014). Budinich *et al.* (2017) studied microbial communities under Pareto optimality to describe all feasible growth rate solutions. Heinken *et al.* (2015a) studied eleven bacteria, along with the human host, to predict interactions under four dietary regimes.

The selection of species is a very challenging step when switching from the investigation of microbial co-occurrences within a large microbiota to the study of functions effectiveness within the community itself. This task entails identifying species of interest among all the microbiota in order to fit various criteria depicting the added value of the microbiome over an individual organism. Along these lines, Julien-Laferrière *et al.* (2016) proposed to extend the gap-filling concept used in metabolic network reconstruction frameworks (Pan *et al.*, 2018) to create synthetic communities among a small number of bacterial candidates. They select species that own enzymes of interest, facultative exogenous genes and model transports between organisms, i.e. exchanged metabolites. The latter can be used either as energy sources or metabolic precursors i.e. building blocks by recipient organisms (Cavaliere *et al.*, 2017). The very high combinatorics between all exchanges viewed as variables makes its impossible to scale their method either to the study of massive datasets involving hundreds or thousands of organisms or to the sorting of possibly hundreds of targeted functions. Eng *et al.* (2016) developed CoMiDA for the purpose of scaling to hundreds of species and targeted compounds using a non-compartmentalized, boundary-free level of modeling, called mixed-bag or gene-soup (Henry *et al.*, 2016). The price to pay for scalability was to get rid of the number of exchanges metabolites involved in the selected community functioning. Although not applied in the same context in terms of the size of datasets considered or the optimization performed, both methods entail optimizing a parsimonious criterion. In one case, the MultiPus algorithm minimizes the total cost of the synthesis, mostly resulting from exchanges and exogenous reactions (Julien-Laferrière *et al.*, 2016), whereas the second algorithm minimizes the size of the community (Eng *et al.*, 2016) and provides one single solution that meets the desired objective. It does not enable sampling nor exploring the possibly broad range of feasible communities that meet the objective used for selection, which can be huge in large microbiotas. This is unfortunate considering that the number of equivalent feasible communities provides hints on redundancy in the microbiome regarding the targeted function, increasing its chances of being really effective. Having several equivalent solutions is an asset, as they can, a posteriori, be filtered by experimenters, based on biological knowledge or additional criteria, prior to experimentation.

In this study, we introduce a method based on the exhaustive enumeration of communities combining a total cost synthesis optimization criterion with a community-size optimization criterion. The tool uses logical programming and combines the non-compartmentalized and the compartmentalized frameworks in order to exhaustively identify and explore all families of species in a microbiota enabling targeted metabolic behaviors. As a first step, the method mimics the approach introduced in Eng *et al.* (2016) to classify a targeted biological function as (i) unfeasible, (ii) feasible with a single organism or (iii) feasible only with several organisms. In the second step, we used logical programming combined with SAT-based solvers (Answer Set Programming) to compute *all* communities with a minimal size allowing a biological function to be effective; this is a generalization of the mixed-bag approach used by Eng *et al.* (2016) in a combinatorial-optimization setting. In the third step, as transports between organisms are costly, we introduce a criterion based on the minimization of exchanges to discriminate between minimal-size community solutions. This entails defining exchangeable metabolites and applying an additional optimization, similar to the one used in Julien-Laferrière *et al.* (2016), to a pre-selected family of communities. This method can be used to facilitate the selection of a community optimizing a desired function in a microbiota by reporting several possibilities which can be then sorted according to biological criteria. The method can also bypass the pre-determination of desired function by being generalized to the screening of all single metabolites within a host-microbial system. An output of the workflow applied in this context is a classification of individual target metabolites in terms of feasibility, functional redundancy and cooperation processes involved.

We applied our workflow to the Human Microbiome Project (HMP) to study community selection for the 4.9 million metabolic functions corresponding to the production of a single metabolic compound (target) in the microbiome from a single metabolic input (seed). Our analysis focused on the distribution of community sizes, redundancy between communities equivalently enabling a function and the complementarity of the mixed-bag and the exchanged-based frameworks. Our approach shows that only 8% of the functions require a community to be enabled, with a maximum of six bacteria. We studied 10% of the latter seed/target functions and observed that in 36.7% of cases, the number of equivalent feasible communities ranges from 100 to 1000 per function, suggesting an significant redundancy of functionalities within the HMP. Using an exchanged-based minimization criteria reduces the family of relevant communities by 24% on average, confirming that both criteria deserve to be considered together. As a matter of application, we investigated the role of different consortia within 773 intestinal bacteria in the production of cytosolic compounds within Recon2.2 human metabolic network.

## 2 Methods and implementation

Given a set of organisms each described by a metabolic model, the goal of our paper is to find a minimal subset of the available organisms that can synthesize a set of target products using available substrates. Our main specificity is that we aim to select the organisms according either to a mixed-bag production of compounds or to a compartmentalized one. The mixed-bag criterion, introduced in (Henry *et al.*, 2016), describes the theoretical capability of a community, considered as a meta-organism, to produce a selected set of compounds thanks to several reactions. However, the use of a reaction by such a consortium involves the exchange of several metabolites: e.g. host gives to the helper one or more reactants that it is able to produce, and the helper gives back the product(s) of this reaction. Thus, reasoning in terms of reactions can be very costly for the organisms when considering what is actually exchanged: the metabolites. This is the main reason for our second compartmentalized criterion, which aims at putting minimization constraints on the metabolites rather than on the number of species. Both criteria are modeled by combinatorial optimization problems, the latter being significantly more difficult than the former because it requires the introduction, as a variable, of all the possible exchanges between all species. We use logical programming to smartly encode a bipartite-based semantics of producibility and solve the combinatorial problems using SAT-based heuristics.

### 2.1 Metabolic models viewed as bipartite graphs

We represent a *metabolic network* as a labeled directed bipartite graph G=(M,R,E), where *R* and *M* are sets of nodes standing for *reactions* and *metabolites* respectively and E⊂M×E∪E×M depict input and output reaction relationships. When (m,r)∈E or (r,m)∈E for m∈M and r∈R, the metabolite *m* is called a *reactant* or *product* of reaction *r* respectively. More formally, for any r∈R, define rcts(r,G)={m∈M|(m,r)∈E} and prds(r,G)={m∈M|(r,m)∈E}.

Seeds S⊆M are compounds that are available to initiate metabolic producibility and fuel metabolic reactions. They usually are the metabolites present in the growth medium of the considered organism, but can also include compounds known to be produced or cofactors in complex internal production cycles (Eng *et al.*, 2016; Greenblum *et al.*, 2012; Julien-Laferrière *et al.*, 2016). Targets T⊆M are metabolites expected to be produced by the considered organism. They can be components (reactants) of the biomass reactions or other compounds, experimentally observed, e.g*.* in metabolomics studies.

The functionality of metabolism is assessed using a producibility criterion. This producibility is calculated using a recursive definition and starts from the seeds. A metabolite is considered producible in two cases: either it is a seed, or it is a product (*prd*) of a reaction *r* whose reactants (*rct*) are producible. Formally, given a metabolic network G=(M,R,E), and a set S⊆M of seed metabolites, a metabolite m∈M is *reachable* from the seeds *S* in the network *G* if m∈S or if m∈prds(r) for some reaction r∈R where all m′∈rcts(r,G) are reachable from *S.* The *scope* of *S* according to *G*, written Σ(S,G), is the closure of metabolites reachable from *S* (Handorf *et al.*, 2005). It is formally defined by $\text{scope}(S,G)=\cup_{i}M_{i}$, where $M_{0}=S$ and $M_{i+1}=M_{i}\cup prds(\{r\in R|rcts(r)\subseteq M_{i}\})$.

The concept of scope has been compared with the flux-based producibility in Kruse *et al.*, (2008). In particular, it is equivalent to the network flow modeling of Eng *et al.* (2016) and to flux-based modeling in general, providing metabolites stoichiometries are set to 1 and accumulation of metabolites is allowed.

### 2.2 Mixed-bag and compartmentalized target producibility

A *community of species* is formally defined as a family of metabolic networks $\left\{G_{1},\ldots G_{N}\right\}$. Following the definition of Henry *et al.* (2016), the mixed-bag or gene-soup modeling of communities considers a boundary-free meta-organism. Organisms’ capabilities are no longer examined individually but collectively in a virtual compartment. Therefore, the associate metabolic network is defined as $\text{mxdBag}(G_{1}..G_{N})=(\cup G_{i},\cup R_{i},\cup E_{i}).$ The scope of the mixed-bag community from a set of seeds *S* is naturally defined as the scope of *S* according to the non-compartmentalized metabolic network: $\text{mxdbagScope}(G_{1}..G_{N},S)=\text{scope}(S,\text{mxdBag}(G_{1}..G_{N}))$.

Considering that all organisms in the community have boundaries and correspond to different compartments requires establishing the family of allowed exchanges between organisms. We denote by exchg(G1..GN)={(rm,i,j)|m∈Mi∩Mj,i=j} the set of reactions enabling the exchange of each compound *m*, which belong to pairs of metabolic networks *G_i_* and *G_j_.* Consider a set of exchange reactions $E\subset \text{exchg}(G_{1}..G_{N})$. The compartmentalized metabolic network associated with the community $G_{1}..G_{N}$ and the family of exchanges E is defined as $\text{cptModel}(G_{1}..G_{N},E)=(\bar{M},\bar{R},\bar{E})$, for which components are compartmentalized with an index: metabolites are denoted by (*m*, *i*), reactions are denoted by (*r*, *j*) and edges are denoted by (*e*, *i*, *j*). More formally, we have $\bar{M}=\cup_{i=1..N}(M_{i}\times \{i\}),\;\bar{R}=E\cup_{i=1..N}(R_{i}\times \{i\})$ and $\bar{E}=\{[(m,i),(e_{m},i,j)]|(e_{m},i,j)\in E\}\cup \{[(e_{m},i,j),(m,j)]|(e_{m},i,j)\in E\}\cup_{i=1..N}\{[(m,i),(r,i)]|(m,r)\in E_{i}\}\cup_{i=1..N}\{[(r,i),(m,i)]|(r,i)\in E_{i}\}$.

**Fig. 1.**
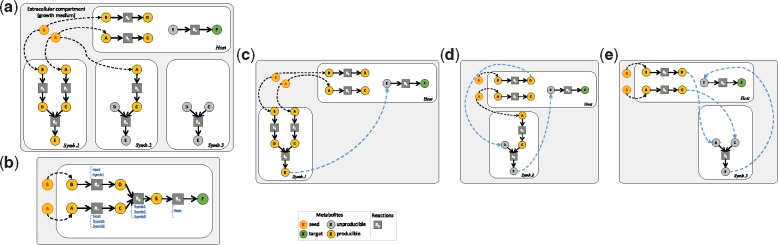
Impact of exchange requirements in community selection, (**a**) Microbiome consisting of a host and three symbionts. Starting from *A* and *B* metabolites as seeds, the objective of the selected community is to produce target *F*, which only the host has the enzymatic capacity for. (**b**) Mixed-bag modeling. Host is the only species owning reaction *R*_3_ to produce *F* from *E.* Symbionts 1, 2 and 3 all possess the *R*_4_ reaction to produce the precursor *E* of the target. The minimal community is of size two and three solutions exist: Host&Symb1, Host&Symb2 or Host&Symb3. Minimal exchanges requirements for all three solutions (compartmentalized modeling) based on the scope producibility criterion are described in sub-figures (c, d, e). (**c**) Exchange of *E* from Symb1 to Host is sufficient for the Host&Symb1 community. (**d**) Host&Symb2 community requires exchange of *D* and *E* metabolites. (**e**) Host&Symb3 community requires exchange of *C*, *D* and *E* metabolites. The unique solution with minimal size and minimal-exchanges is Host&Symb1

Given a set of substrate metabolites *S*, we allow each of the seed in *S* to be imported into each considered organisms by creating a set of compartmentalized seeds: $\text{cptSeed}(G_{1}..G_{N},S)=\cup_{1..N}(S\cap M_{i}\times \{i\})$. As shown in Figure 1, the media compounds *A* and *B*, which both belong to the cytosolic metabolic networks of host and symbiont 1, are considered their seeds. Symbiont 2 has only *A* as seed and symbiont 3 has no internal seeds. By extension, we say that a target *t* belongs to the scope of the compartmentalized metabolic model if there is a metabolic model *G_k_* that contains *t* and has it in its scope: $\text{cptScope}(G_{1}..G_{N},E,S)=\{t\in \cup_{1..N}M_{i}|\exists k,(t,k)\in \text{scope}(\text{cptSeed}(G_{1}..G_{N},S),\text{cptModel}(G_{1}..G_{N},E))\}$. Based on this definition, in Figure 1, the compound *E* belongs to the scope of {*A*, *B*} because it can be produced in symbiont 1. On the contrary, target *F* does not belong to the compartmentalized scope of {*A*, *B*} even though it belongs to the mixed-bag scope of {*A*, *B*}. There is not way of producing the precursor *E* in the host species according to the compartmentalized metabolic network: a transport of *E* to the host is needed.

Given a set of compounds *T* considered as relevant targets, we define a set of exchanges E to be consistently associated with a mixed-bag community in agreement with *T* when it makes it possible to produce, in the compartmentalized model, all the compounds from *T* that used to be producible in the mixed-bag model, i.e.: $T\cap \text{cptScope}(G_{1}..G_{N},E,S)=T\cap \text{mxdbagScope}(G_{1}..G_{N},S)$. Considering the producibility of metabolites for deciphering exchanges makes it possible to discriminate between models. In Figure 1, the three symbionts possess a reaction of interest that would enable the producibility of the target by the host when adding transports.

### 2.3 Mixed-bag selection of community

Selecting a sub-community following the mixed-bag model entails picking a minimal number of organisms, such that they can collectively meet an objective regardless of transport reactions and exchanges. The search space C is the set of the $2^{N}$ sub-families of the whole community. We select organisms in the mixed-bag community to be added to an empty system (or a system with a host) such that a maximum of its targets are producible, by maximizing the size of $T\cap \text{mxdbagScope}(G_{i_{1}}..G_{i_{L}},S)$ among all possible sub-communities $\left\{G_{i_{1}}..G_{i_{L}}\}\subset \{G_{1}..G_{N}\right\}$. This selection of reactions must occur in a minimal number of organisms, hence the minimization of the number of species *L* in a second step. Optimal community is defined by successively solving these two problems:

mxdbagCnity(S,T,G1..GN)=arg min{Gi1..GiL}⊂{G1..GN}(size (T∖mxdbagScope(Gi1..GiL,S)), size {Gi1..GiL}.

For instance, in Figure 1 (a, b), there are three different ways to produce target *T* from the medium {*A*, *B*} according to a mixed-bag framework: the host can be combined with any of the three symbionts 1, 2 and 3, which all possess the reaction *R*_4_, producing *E* from *C* and *D.*

As the mixed-bag formalism is subjected to a small amount of constraints due to the absence of transport or exchanges modeling, it can be used as a first study of the community and to pinpoint members of interest for experimenters. Eng *et al.* (2016) implemented CoMiDA, an Integer Linear Programming (ILP) algorithm for solving this problem with a network-flow formalism. They tested it on 10 000 random pairs of seed and target singletons to identify a minimal community. However, CoMiDA, like the other problem-solving techniques, proposes a single solution to the gene-soup sub-community problem, thus preventing the experimenter from catching the global and possibly complex combinatorics of the problem. Topological modeling with Answer Set Programming (ASP; Gebser *et al.*, 2012) has solving assets, as it enables us to sample the whole space of the solutions and thus provide enumeration of optimal solutions or their intersection-union. The foundations of an ASP-encoding enabling the identification of optimal communities is depicted in Listing 1.

**Listing 1**

Selection of minimal-size communities in a mixed-bag framework

- {sel_orga(B): orga(B)}.
- mixedbagScope(M):- seed(M).
- mixedbagScope(M):- sel_orga(B); product(M, R); reaction(R, B); mixedbagScope(M2): reactant(M2, R).
- #minimize {1@2, T: target(T), not mixedbagScope(T)}.
- #minimize {1@1, B: sel_orga(B)}.

### 2.4 Exchange-based selection of community

The mixed-bag modeling should be considered as a method for globally studying the metabolic complementarity of community members. A limitation of the mixed-bag modeling is that it does not take into account the required exchanges needed when setting back the boundaries of the metabolic models. A natural motivation is that it is energetically demanding to export or import metabolites, hence a parsimonious hypothesis of exchange dependencies in organisms. Julien-Laferrière *et al.* (2016) have introduced an algorithm for the selection of synthetic community based on the minimization of exogenous reactions and transport reactions. Without much information on precise transportable mechanisms, as it is the case for many non-model organisms, the size of the search space consisting of all possible exchanges is 
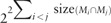
. This makes the search for minimal exchanges an intractable problem for microbiomes with a high number of organisms, and particularly if their models contain a large set of shared metabolites. This occurs a lot in models that are built automatically from metagenomics as they cannot be individually curated and improved.

To solve this issue, we introduce a heuristic to approximate the exchange minimization problem, which entails filtering minimal size communities with criteria based on the number of exchange compounds. This is modeled using an optimization problem chaining three combinatorial optimizations: maximizing the number of produced targets in the community under mixed-bag assumption (as seen before), minimizing the size of the community (as seen before) and, then, minimizing the number of exchanges by considering organisms boundaries again. Therefore, the family of optimal communities in the compartmentalized setting is formally defined as follows:

cptCnity(S,T,G1..GN)=arg min{Gi1..GiL}⊂{G1..GN}(size(T∖mxdbagScope(Gi1..GiL,S)),size{Gi1..GiL},size{E⊂exchg(Gi1..GiL)|T∩cptScope(Gi1..GiL,E,S)=T∩mxdbagScope(Gi1..GiL,S)}.

Such optimal communities with their associated minimal sets of exchanges can be identified using the ASP programming paradigm with an extension of the previous encoding, and applied to communities pre-selected with the mixed-bag framework. The predicate *escope* is introduced to recursively compute the scope of the compartmentalized model associated with a selected family of organisms and exchange reactions (Listing 2).

**Listing 2**

Selection of minimal-size communities in a compartmentalized framework, together with a minimal size of exchange reactions

- {sel_orga(B): orga(B)}.
- {exchanged(M, O1, O2): metabolite(M, O1); metabolite(M, O2); escope(M, O1); sel_orga(O1); sel_orga(O2); O1!=O2}.
- escope(M, O):- seed(M); sel_orga(O).
- escope(M, O):- exchanged(M,_, O), sel_orga(O).
- escope(M, O):- product(M, R); reaction(R, O); sel_orga(O); escope(M2, O): reactant(M2, R).
- #minimize{1@3, M: target(M), not escope(M,_)}.
- #minimize {1@2, B: sel_orga(B)}.
- #minimize{1@1, M, O1, O: exchanged(M, O1, O)}.

This approach discriminates the three solutions obtained with the mixed-bag modeling in Figure 1. Cooperation between the host and symbiont 1 requires the latter to provide the compound *E* (that belongs to the individual scope of symbiont 1) to the host in order to activate the production of *F.* Alternatively, the host can be combined with symbiont 2 by providing it the *C* compound in order to produce *E*, which can be transferred to the host in return. Finally, the host can be combined with symbiont 3, by providing it with *C* and *D* compounds in order to produce *E*, which activates the production of *F.* The host-symbiont 1 community is the optimal one to restore producibility of *T.*

### 2.5 A workflow for the target-based screening of a microbial consortium associated with a host

The Miscoto Python tool (MIcrobiome Screening and COmmunity selection using TOpology) encapsulates the previous ASP encodings and takes as input a family of metabolic networks each corresponding to a symbiont, a metabolic network associated with a main species called *host* (optional), a set of seeds depicting the medium compounds and a set of targets depicting the expected products. For the sake of an exhaustive target study, the family of targets can be set to be equal to all cytosolic compounds of the host, or the whole set of compounds in the microbiome and host.

**Fig. 2.**
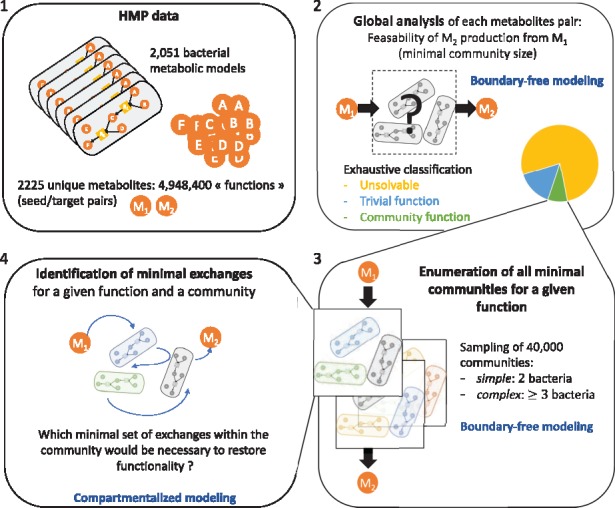
Workflow applied to the HMP: 4 948 400 pairs of seed/target metabolites (i.e. functions) were tested according to their capability to be produced from the microbiome. (i) *Function feasibility*: pairs were classified in three categories: unsolvable, trivial or community functions. (ii) *Minimal community size computation*: the minimal size of the community allowing us to restore the producibility of 40 000 community functions was computed. (iii) *Exhaustive enumeration of minimal communities*: 5301 pairs that required a community of three or more bacteria were explored in more depth by enumerating all the possible minimal communities allowing us to restore the producibility. (iv) *Sorting according to minimal exchanges*: for the 5301 pairs, a minimal set of exchanges explaining the predicted cooperation was computed. The complete set of minimal exchanges was computed for all communities obtained for a subset of complex functions

The first step of the analysis of the microbial consortium (Fig. 2) is a *feasibility analysis.* It entails identifying the added-value of the symbionts for the production of metabolic compounds of the system. Targets are classified according to three criteria: *unsolvable target function* (the target is not producible by the host associated with all its symbionts)—*trivial target function* (the target is producible by the host or, if no host is provided, a single species of the community)—*community target function* (the target is producible by the host only when it is combined with one or more symbionts in the community or, if no host is provided, two or more symbionts). To that end, a mixed-bag framework is sufficient.

As a second step, for each community function, the algorithm depicted in listing 1 allows for the *computation of the minimal size of the community* required to activate the function. The third step is an *estimation of functional robustness.* Intuitively, we can expect that the more complex (poor distribution of enzymes of interest among the bacteria, large set of targets) the metabolic objective is, the lower the number of minimal communities there should be to activate a function within a microbial community. In addition, enumerating solutions in synthetic community design can provide experimenters with alternatives for countering biological incompatibilities between bacteria (Julien-Laferrière *et al.*, 2016). To that end, reasoning-modes of ASP are used to estimate the functional robustness associated with a family of selected functions: first, the union of species involved in at least one minimal community required to activate a function is easily computed with a brave enumeration mode to estimate the range of redundancy associated to the function. All minimal communities associated with the targeted function can then be enumerated if needed.

The last step is related to the *identification of minimal exchange communities* among the ones previously identified in step 2. Minimal-exchange communities can also be enumerated for a biological-expert analysis. This allows us to select communities with a minimal number of required transports in order to decrease the expert-based analysis of exchanges needed to assess their biological feasibility.

Performances depend on the complexity and amount of species involved in the experiment. As an example, the first step combined with the enumeration of minimal-size communities (mixed-bag) lasted around 300 s for the study of Recon and the 773 bacteria described in the results on a personal computer. The combination of the second and third steps lasted 100 s in average for each individual target. As runs are independent they can easily be parallelized. Finally, the identification of minimal exchanges (and their union) lasted around 200 s per target, less than 4 h for all targets combined in one run.

Notice that a key point for the applicability of the workflow is that it relies on a three-level modeling strategy: feasibility is based on a scope computation within a global metabolic model; functional redundancy is addressed with a mixed-bag metabolism in which all metabolic enzymes are shared and exchanges are costless; cooperation processes rely on a compartmentalized framework. This strategy is motivated by computational issues: a direct computation of relevant communities by minimizing both the number of bacteria and the number of metabolic transports among them is not conceivable due to the high combinatorics of the problem. For instance, in the HMP benchmark studied in the Results Section, due to the large overlap of metabolites in the 2051 models integrated into the benchmark, examining exchanges during the community selection step without any a priori on the bacteria requires considering $2^{3.10^{9}}$ possible transports and makes it impossible to identify minimal communities. On the other hand, considering that exchanges can occur in communities without computing them as a first-line objective enables us to globally analyze the benchmark.

## 3 Results

### 3.1 Bacterial complementarity in the HMP

#### 3.1.1 Design of the analysis workflow

The HMP Consortium stool sample (Human Microbiome Project, 2012) was the first large dataset to describe the abundance and variety of bacterial functions in the gut. It represents a valuable resource for exploring microbial cooperation in the gut metabolism. Eng *et al.* (2016) produced metabolic models for the HMP data. Each of the 2051 bacteria has its own metabolic model, the union of all consisting of 3606 unique reactions. The average size of each metabolic model is 1096 reactions. A benchmark was established to validate their flow-inspired algorithm by selecting a minimal community of bacteria to produce a target metabolite from a seed one. Ten thousand random pairs of two metabolites (one seed, one target) were randomly tested. The authors used a ILP algorithm and several formalisms, of which the most constrained was a bipartite graph modeling, equivalent to the mixed-bag framework introduced in Section 2.2. The goal of the algorithm was to enable the production of the targeted compound, assuming that the seed was considered to be the only available compound for the bacteria in the consortium to activate their reactions. The authors showed that species could be selected to produce the desired target from the unique seed in less than 5% of the cases in the benchmark, thus concluding that in most cases among the ones tested, no path exists between the seed and the target.

Miscoto allowed the analysis of Eng *et al.* (2016) to be pursued by designing a pipeline that enables an exhaustive exploration of the previously benchmarked dataset (available on demand to the authors). The pipeline is depicted in Figure 2. We first calculated the metabolic feasibility by a microbial community of all possible 4 948 400 seed/target pairs of metabolites, which we call *functions.* We then studied the functional redundancy by sampling functions associated with a community—i.e. requiring at least two bacteria to be met—and exhaustively enumerated all minimal solutions for each function that required at least three bacteria. Ultimately, we focused on cooperation processes and showed that exchange computation can discriminate between size-minimal communities by identifying minimal metabolic exchanges required within the selected bacteria.

#### 3.1.2 Exhaustive feasibility study of the HMP seed/target functions

**Fig. 3.**
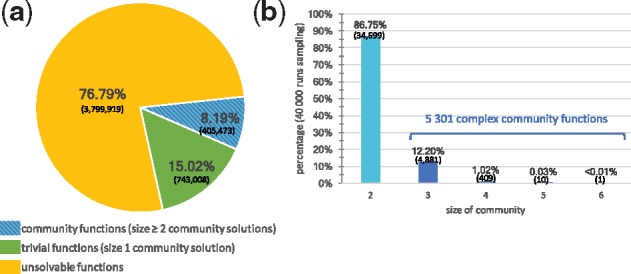
(**a**) Exhaustive study of the feasibility for the 4 948 400 seed/target functions associated with the HMP dataset. (**b**) Community size computation for 10% of community functions (i.e. 40 000 seed/target pairs associated with a community of size 2 or more): all communities have size less than 6, the most frequent case being size 2

Figure 3 depicts the results of the exhaustive study of feasibility of all of the 4 948 400 functions in the HMP dataset, that is, all possible pairs combining any single seed from the microbiota compounds associated with any single target from the microbiota compounds. We classified functions in three categories: unsolvable functions that can never be reached regardless of the selected community, trivial functions that can be met by a single bacterium and community functions that can only be met through bacterial cooperation (two or more bacteria). Our analysis demonstrated that 76.8% of functions are unsolvable in the benchmark, which is expected, as we only allow one metabolite to initiate metabolic reactions, i.e. the unique seed. We notice however that feasible functions among the complete family of seed/target functions are five times more frequent than estimated in the random benchmark of Eng *et al.* (2016), militating in favor of exhaustive studies of feasibility with a complete view of the producibility capabilities of microbiomes. Our tool also evidenced that 15.0% of the functions are trivial i.e. intrinsically met in one metabolic model. The remaining 8.2%, or 405 473 functions, depend on two or more bacteria: they are community functions (Fig. 3a). This analysis illustrates that Miscoto can efficiently classify functions by ensuring an exhaustive exploration of a large-scale microbiome and point out complex community functions.

To have a better insight of the size of the community associated with community functions, we randomly selected 10% of them (40 000 community functions) and used both our Miscoto tool and the network-flow CoMiDA algorithm (Eng *et al.*, 2016) to identify a minimal community of bacteria that enables us to complete them. Both tools reported similar results, confirming that the scope-based semantics encoded in Miscoto and the bipartite-graph model implemented in CoMiDA are equivalent. We observed that 86.8% of community functions are simple: two bacteria cooperating are enough to restore the function (Fig. 3b). The maximal size of communities needed to restore a function is equal to 6. There are 5301 (13.2%) complex community functions of size three or more, with most of the functions depending on three bacteria (12.2% of the benchmark, i.e. 4881 functions), and 420 being of size 4 or more. Together, this analysis suggests that a very low percentage (1% provided that the sampling of complex community functions was representative, at most 7% in all cases) of the whole set of seed/target functions is made feasible with communities of three or more bacteria.

#### 3.1.3 Exploring the whole space of solutions demonstrates functional redundancy of microbiota metabolism

**Fig. 4.**
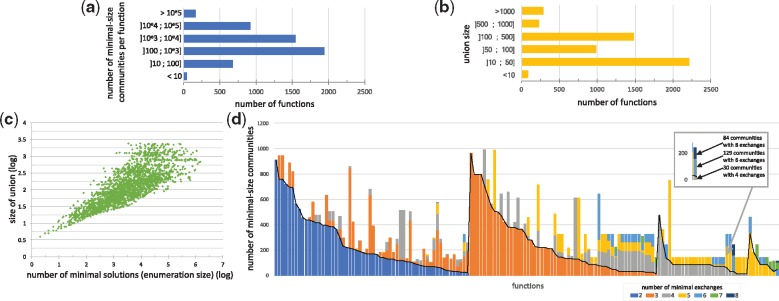
Minimal-size solutions (communities) for each of the 5301 complex functions associated with three or more bacteria were enumerated.(**a**) Distribution of the enumeration size. (**b**) Distribution of the number of bacteria involved in at least one solution (union size). (**c**) Relationship between size of union and number of solutions. (**d**) Minimal exchange communities. Seed/target functions of 150 associated with less than 1000 minimal communities of three bacteria were selected. For each minimal community (53 081 in total), the minimal number of exchanges required to make effective the target producibility in a compartmentalized framework was computed. Each vertical bar stands for a seed/target function. Colors depict the number of minimal communities associated with a set of exchanges of a given size for each function. As an example, the producibility of the C13629 target from the C00214 seed is made feasible by 243 communities of three bacteria. 30 of these communities have a minimal number of 4 exchanges, 129 communities of 6 exchanges and 84 communities of 8 exchanges. Focusing on communities with minimal-exchanges (below the black line) reduces the total number of communities by a ratio of 45% and the size of the union of bacteria involved in solutions by 24%

We used the enumeration capability of ASP-based methods implemented in the Miscoto tool to enumerate minimal community solutions for all complex functions (associated with three or more bacteria) identified in the 40 000 functions sample, i.e. 5301 ones. The number of minimal communities per function ranged from 2 to 1 506 662. As shown in Figure 4a, 86.5% of functions generated more than 100 solutions, of which 49.8% generated more than 1000 solutions. The median is 977 solutions per function. This illustrates the high combinatorics of the community solving problem. In terms of biological interpretation, the number of minimal communities associated with a function can be regarded as a pointer to functional redundancy: a function associated with a large number of minimal community solutions in the microbiome is more likely to be effective than one with a very few number of solutions because several bacteria are able to play an equivalent role with respect to the function restoration (Moya *et al.*, 2016). To confirm this hypothesis, for each seed/target function in our sample, we computed the number of bacteria involved in at least one minimal community restoring the function, i.e. the union of bacteria (Fig. 4b). Our analysis suggests that the number of solutions is linked with the number of species involved in a solution with a polynomial relation (Fig. 4c). In this respect, computing the latter is much less time-consuming than the former and is easier to study than a large set of enumerated solutions (Fig. 4b) as the median size of the union is 68.5. This promotes the use of the quantity of bacteria involved in at least one minimal solution to the function restoration problem. When screening a large set of targeted functions, this criterion enables us to sort functions according to their redundancy and determine whether it is worth enumerating the whole set of minimal communities.

#### 3.1.4 Identification of exchanges to discriminate minimal-size communities associated with a given function

In order to get a better insight into the variability of cooperation processes involved in minimal-size communities, we further developed the analysis by selecting a panel of 150 functions for which the minimal size of solution was three bacteria and which displayed a number of community solutions ranging from 100 to 1000, which is the main range identified in the enumeration study. We noticed that the number of minimal exchanges ranges from 2 to 8 for communities optimizing the producibility of one function (Fig. 4d). For 94% of all functions, there is at least one community solution with at most four exchanges. 38.7% of the functions can be met by at least one community associated with two exchanges only: they correspond to the simplest exchanged-based functioning for a community of three species, where two bacteria each provide a single precursor to a third one. The functioning of 60.9% of the remaining functions can be explained with three exchanges, suggesting that exchanges within several bacteria (e.g. the production of two precursors by a bacterium or a cycle system between two bacteria) are needed to make the function effective. The other cases correspond to more complex cases with multiple exchanges within the consortium.

As shown in Figure 4d, only 17% of the studied functions are associated with minimal-size communities that all depict the same number of exchanges (monocolor vertical bars in the picture). For the 83% non-homogeneous functions, focusing on communities with a minimal number of exchanges reduces the number of communities to be explored by 45%; these minimal-exchanges communities are depicted with the lowest color segment in each vertical bar. Focusing on these minimal-exchange communities allows us to reduce the average family of bacteria (union) involved in the possible communities from 43 to 30. This suggests that adding an exchanged-based criterion to the community-size optimization criteria may facilitate the selection of strains associated with a targeted function by reducing the number of relevant species to be investigated closely.

### 3.2 Recon 2.2 and gut microbiota complementarities

For the sake of application and illustration, the Miscoto tool was applied to study cooperation potential between the Recon 2.2 genome-scale metabolic model of the human metabolism (Swainston *et al.*, 2016) and 773 gut microbial models [Magnúsdóttir *et al.* (2016), available on: vmh.uni.lu]. In order to globally analyze and screen the cooperation within these organisms under fixed nutritional conditions, nutrients were voluntarily restricted to 51 compounds of the Dulbecco’s Modified Eagle’s growth medium (DMEM). This medium mimics a type of cell culture conditions that can suit enterocytes (intestinal absorptive cells) and has been shown to enable some bacterial growth (Biedermann *et al.*, 2014).

We set all cytosolic metabolites of Recon as metabolic targets, i.e. a set of 1920 compounds, having in mind that this target list could be refined in the future, following an intestinal version of the human metabolic network. We noticed that 831 compounds are producible from the DMEM growth medium, whereas 1451 metabolites are producible from native modeling conditions of Recon2.2 using as seeds all boundary compounds for which imports to the extracellular space and cytosol exist. A feasibility analysis with Miscoto highlighted that cooperation with the gut microbes may facilitate the producibility of 46 additional target metabolites with the DMEM growth medium and 24 with the native modeling conditions of Recon. This confirms that the medium is very restrictive and that bacteria have an increased added value on the host in a limited growth medium rather than in the native modeling conditions.

The minimal-size community identification implemented in Miscoto evidenced that, in cooperation with Recon, 381 different communities of three bacteria are enough to enable the producibility of the whole set of 46 targets. Between 42 and 48 exchanges may be needed to make this producibility effective, mostly from the bacteria to the host. Only 11.5% of the gut microbiome, that is to say 89 bacteria, play a role in the at least one of these 381 communities with a cooperation potential. We identified a unique partition of the 89 bacteria in three disjoint clusters of 58, 15 and 16 species such that each of the 381 minimal communities comprises exactly one species from each cluster. We noticed however, that the bacteria are far from playing equivalent roles within each cluster: each species in a cluster is associated with only few species in the two others. This suggests that bacteria do not have equivalent roles regarding the individual producibility of targets.

**Fig. 5.**
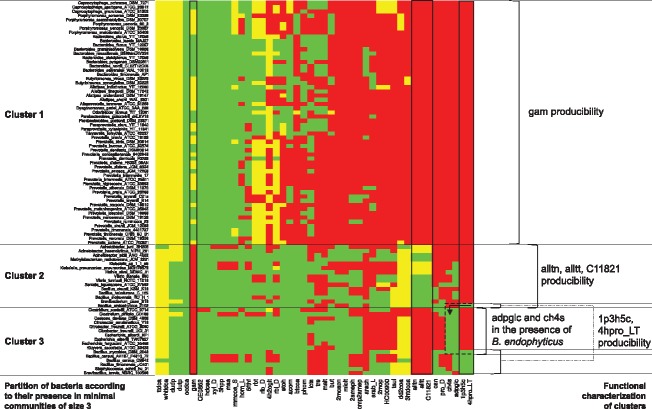
Feasibility analysis of a family of 89 bacteria (lines) involved in optimal cooperations with Recon according to the producibility of 46 Recon cytosolic compounds (columns, identifiers from the BiGG database). Bacteria enabling the producibility of the corresponding target are depicted by a green spot when the required number of exchanges is minimal among all bacteria associated with the target. A yellow spot describes the case when the number of associated exchanges is not minimal with respect to the considered target. Red spots depict bacteria which do not restore the considered target producibility. The 89 bacteria are partitioned into three clusters such that each of the 381 optimal communities enabling the producibility of the whole set of 46 targets comprises a bacterium in each cluster. These clusters can be discriminated by the producibility of eight compounds

In order to elucidate both the role of the three clusters of bacteria and the individual role of species in each group, we screened the impact of the 89 bacteria over the 46 individual targets with Miscoto. The feasibility analysis highlighted that the producibility of each individual target can be restored by at least a single bacterium. Figure 5 depicts the connection between each target and each bacterium. It highlights that some targeted compounds can be produced by only very few species, leading to further insights into the 381 optimal communities identified by the Miscoto tool. A first discriminating compound to produce is D-glucosamine (*gam*), which can only be produced by the species from the Cluster 1 (mostly *Prevotella*, *Bacteroides* and *Porphyromonas* bacteria). The second discriminating family of three compounds involving allantoin (*alltt, alltn, C11821*). They can only be produced by species from the Cluster 2. A third family of two discriminating compounds is related to the hydroxy-proline producibility (*X1p3h5c* and *4hpro_LT*): they can either be produced by a sub-family of Cluster 3, or by *Bacillus endophyticus* from the Cluster 2. In the latter case, ADP-glucose (*adpglc*) and methanethiol (*ch4s*) have to be produced by a sub-family of Cluster 3.

Provided that these eight compounds are made producible, the producibility of the 38 remaining compounds in ensured by restricting to some homogeneous groups of bacteria (*Porphyromonas*, *Vibrio*, *Capnocytophaga*, *Allistipes*, *Paraprevotella*, *Citrobacter*, *Escherichia*, *Bacteroides*, *Prevotella*) which have similar impact over the producibility of the 46 targets (their associated lines are identical). Some groups represented by a large number of strains may have few differences in terms of target production, possibly explained by gaps in metabolic networks due to differences in genome annotation. Nevertheless, these differences have no impact on the selection of optimal communities. Taken together, this analysis illustrates that the role of the different bacteria in the production of a multi-target function by an optimal community can be elucidated by using the screening of individual target feasibility with the Miscoto tool.

## 4 Discussion

Selecting communities in large microbiotas has a large spectrum of applications ranging from the understanding of complex eukaryotes dependencies to their symbionts to the design of synthetic communities for industrial applications. In any case, having access to the whole of systems satisfying the desired objective is an asset, should additional criteria or biological incompatibilities be taken into account a posteriori. In this paper, we presented the Miscoto tool and its use to perform a wide and efficient screening of microbiomes in the context of community selection by combining size and exchange combinatorial optimizations. Miscoto enables the classification of metabolic producibility in terms of feasibility, functional redundancy and cooperation processes involved. By providing, as a first step, a feasibility analysis in the microbiota, it can assess the global added value of any microbial cooperation with regards to a target producibility objective. Identified community functions will require cooperation to occur, either between a host and at least one symbiont, or within a group of two or more bacteria. These functions can be further analyzed to identify all minimal-size communities that can meet them. We showed that the union of symbionts that appear in at least one solution is helpful for determining the redundancy of the function in the microbiome. The following step of exchange minimization within minimal-size communities forms a means for classification of these communities. The application of this workflow to the HMP data constitutes, to the best of our knowledge, the first exhaustive and large scale analysis of cooperation capabilities in a microbiome dataset.

A first key feature of the method is to address scalability in large datasets by minimizing the size of the community prior to the exchanges. Let us emphasize that our approach is particularly suited for screening large-sets of bacteria and compounds. Once the family of bacteria has been drastically reduced, it becomes possible to skip the minimal-size criteria and directly run the exchange minimization-based criteria to have an exhaustive view of relevant exchanges within a small-scale community. A limitation of the method could occur in some cases, e.g. the selection by Miscoto of a 2-bacteria community with 3 minimal exchanges while there also exist a 3-bacteria community with 2 exchanges.

A second key feature of the method is that it entirely relies on a combinatorial modeling of metabolism based on bipartite graphs. The producibility is checked according to a recursive definition of the propagation (scope) which does not take into account the stoichiometry of reactions. This choice was motivated first for computational reasons, since the combinatorial framework makes the producibility criteria monotonous: adding a reaction to a network can only increases the scope (Prigent *et al.*, 2017). The enumeration of all solutions is performed in a reasonable time with SAT-based techniques. Notice, however, that is is possible to filter a posteriori the family of reported communities with flux-based criteria.

As a last feature, our framework does not take into account the potential competition, incompatibilities in cultivating the proposed bacteria together as raised by Julien-Laferrière *et al.* (2016), nor the possibility that key metabolites could be degraded (Eng *et al.* (2016)). The ASP paradigm we use is flexible enough to include any knowledge about strain compatibility. However, we consider that not all experimental parameters can reasonably be formalized in such large scale datasets. Therefore, for the sake of genericity, we advocate for the identification of all optimal models without a priori considering experimental knowledge. Furthermore, metabolic transport identification is a challenge in metabolic models that highly depends on genome annotation quality. There usually are few metabolites for which transport between the cell and the extracellular space is assessed in non-models species without an extensive review of the literature (Sung *et al.*, 2017) although efforts are made to compute them automatically (Dias *et al.*, 2017). Community solutions that would require unexpected transports can be discarded a posteriori. For others, an additional optimization is an option, by prioritizing exchange of metabolites for which transport is identified in the metabolic models. More generally, the use of any criteria that are context-specific can be done a posteriori to rank the communities to be tested without leaving any optimal solution aside. In particular, such considerations have to be acknowledged when selecting a subset of a microbiome for experimental verification that a function performed by the host requires cooperation.

In addition to the selection of microbial communities, let us point out that the search for exchange interactions can also be of strong use in the process of metabolic network reconstruction and genome annotation, particularly when it is unclear whether a host can carry some metabolic functions by itself or if it relies on its symbionts to do so (Dittami *et al.*, 2014). Gap-filling steps performed in model reconstructions risk overfitting when they do not systematically propose associated genes to the added reactions (Pan *et al.*, 2018). This raises questions related the existence of some associated functions in the organism compared to its dependency on an obligate symbiont to execute it. Such reactions are included to meet a defined objective but in a context in which the organism is self-sufficient to meet it, which is a substantial hypothesis when facing difficulties with growing a non-model organism in axenic conditions. This shows the need to work with possibly incomplete models and, if possible, check the added-value of the microbiome before adding putative reactions with no genetic genetic support to restore functions (e.g. biomass production).

To conclude, we see our community selection workflow as an intermediary step in microbiome studies prior to quantitative constraint-based simulations (Hanemaaijer *et al.*, 2017; Heinken *et al.*, 2015a; van der Ark *et al.*, 2017) in order to handle the combinatorics of community selection in large microbiotas.
